# Successful Cardiopulmonary Resuscitation in Pregnancy: A Case Report

**DOI:** 10.4021/jocmr2010.02.257w

**Published:** 2010-02-16

**Authors:** Ozgur Sogut, Atilla Kamaz, Mehmet Ozgur Erdogan, Yusuf Sezen

**Affiliations:** a Department of Emergency Medicine, Harran University, School of Medicine, Sanliurfa, Turkey; b Department of Anesthesiology and Intensive care medicine, Kahramanmaras state hospital, Kahramanmaras, Turkey; c Department of Emergency Medicine, Sanliurfa Research and Training Hospital, Sanliurfa, Turkey; d Department of Cardiology, Harran University, School of Medicine, Sanliurfa, Turkey

## Abstract

The management of cardiac arrest in pregnancy is an important task for the emergency physicians. The clinical outcome of mother or fetus will often depend on the successful resuscitation of the first few minutes. Furthermore, the resuscitation team leader should consider the necessity of emergency hysterotomy (cesarean delivery) as soon as a pregnant woman develops cardiac arrest. We report a case of a 28-year-old pregnant woman who had a ventricular fibrillation cardiac arrest. She was successfully resuscitated in our emergency department and a single male healthy infant was delivered via cesarean section at 36 weeks’ gestation. Mother and baby were discharged survival and neurologically intact from the intensive care unit (ICU) on day 25. We emphasize that understanding the causes of cardiac arrest during pregnancy, its early recognition and prompt resuscitation by recent ACLS guidelines may decrease both maternal and fetal morbidity or mortality.

**Keywords:**

Cardiac arrest; Cardiopulmonary resuscitation; Cesarean delivery; Fetus; Pregnant

## Introduction

Cardiac arrest during pregnancy is an infrequent event. The incidence of cardiac arrest has been reported as 1 in every 30.000 near-term pregnancies by the Resuscitation Council (UK) [1]. Cardiac arrest with pregnancy has numerous causes. Major causes include amniotic fluid embolism, hemorrhagic shock, eclampsia, pulmonary thromboembolic events or sepsis [2]. Anaphylaxis, trauma, congenital or acquired cardiac diseases are the minor causes of cardiac arrest during pregnancy [2,3]. The clinical outcome of mother or fetus with cardiac arrest in pregnancy will often depend on the successful resuscitation of the first few minutes [4]. There have been a few number of reported cases with successful resuscitation of both mother and baby after cesarean delivery [4,5].

Herein, the authors report a case of sudden cardiac arrest caused by acquired valvular cardiac disease in a 36 weeks pregnant young woman who was successfully resuscitated in the emergency department (ED). The patient and infant were discharged survival and neurologically intact from the intensive care unit (ICU).

## Case Report

**Figure 1:**
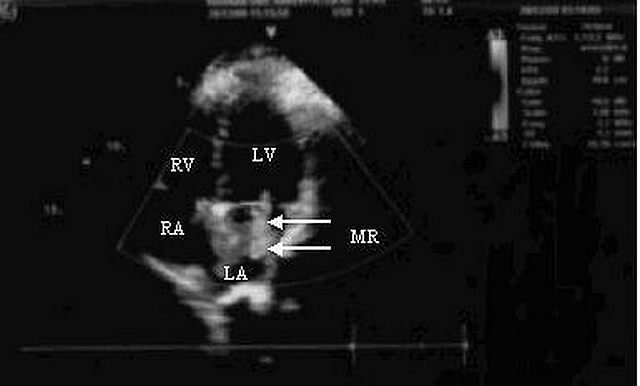
Transthoracic echocardiogram (TTE) showing moderate to severe mitral regurgitation (MR) with an estimated regrgitant fraction of 45% (white arrows).

A 28-year-old pregnant woman was found unconscious in bathroom of her house by her husband. At arrival in the ED, she had a Glasgow Coma Score of 3/15 and dilated sluggish pupils. Additionally, there were no heartbeats or respiration. Cardiopulmonary resuscitation (CPR) was immediately instituted and cardiac monitoring revealed ventricular fibrillation. She was treated with 200 J of direct current energy delivered twice. Intravenous epinephrine (1 mg) was administered after the second rescue shock. Neither atropine nor antidysrhythmic agents were administered. After 2 minutes of CPR, her rhythm has restored to sinus rhythm. Advanced cardiac life support measures were initiated (such as controlled intravenous fluid support, administration of dopamine, adequate oxygenation, and support of respiration with bag ventilation) in ED. There was no tobacco, alcohol or illicit drug use in her history. Her husband stated that she had a brief episode of discomfort palpitations and shortness of breath three weeks prior to her cardiac arrest. However, there was no history of cardiac or respiratory disease. Transabdominal ultrasound confirmed 36 weeks pregnancy with fetal cardiac activity. Noncontrast cranial computerized tomography was nondiagnostic. Laboratory studies taken on admission and urine drug screen were not remarkable. A bedside emergency transthoracic echocardiogram (TTE) demonstrated moderate to severe mitral regurgitation with an estimated regurgitant fraction of 45% (Fig. 1). Additionally, there was no hypokinesis, effusion or ventricular dysfunction. During her emergency department course, the patient began to response from painful stimuli. Propofol was initiated for sedation. Three hours after admission to the ED, she was transferred to the ICU for optimal care. Support of respiration with mechanical ventilation and general supportive measures were provided in ICU. The clinical course of the patient was uneventful. She did not require vasopressors or antidysrhythmics during her ICU course. On the sixth hour of admission, she was declared hemodynamically stabilized and cesarean section was performed. Single male healthy newborn was delivered. Sedation was subsequently discontinued and the patient awoke on hospital day 5 with mild neurologic deficit. On the 15th day of admission, the decision was made to extubate the patient. Mother and baby were discharged survival without neurologic deficit on ICU day 25.

## Discussion

The anatomic and physiologic changes during pregnancy result in significantly decreased cardiovascular and pulmonary reserves which complicate resuscitation [6]. During attempted resuscitation of a pregnant woman, providers have two potential patients, the mother and the fetus. Correct and fast decision of resuscitation team leader is essential in the maternal and fetal survival. The best hope of fetal survival is maternal survival [2]. The rescuers should be kept in mind that you will lose both mother and infant if you cannot restore blood flow to the mother’s heart [7]. In this case, maternal successful resuscitation supported the fetal survival.

Mitral regurgitation (MR) is the most common acquired valvular cardiac disease in women [8]. Pregnant patients with valvular regurgitation (such as mitral regurgitation or aortic regurgitation) are low-risk organic cardiac lesions and they tolerate pregnancy well. Cardiovascular collapse may rarely seen in pregnants with acute MR [3,8]. In this patient moderate to severe MR was diagnosed via a bedside TTE in ED. We suspected that MR with decreased cardiovascular and pulmonary reserves during pregnancy may lead to develop ventricular fibrillation arrest in this patient.

The resuscitation team leader should consider the need for an emergency hysterotomy (cesarean delivery) protocol as soon as a pregnant woman develops cardiac arrest [7,9]. Advanced Cardiac Life Support (ACLS) guidelines recommend that if the mother’s pulse has not been restored within 4 to 5 minutes, perimortem cesarean section should be performed. Fetal viability must be considered in this decision [10].

In this case, CPR did not last more then two minutes so there was no need for emergency hysterotomy. Fetal assessment starts with measurement of fetal cardiac activity to establish fetal viability. Fetal viability begins at approximately 24 to 25 weeks [11]. Portable ultrasonography, available in some emergency departments, may aid in determination of gestational age. This step is recommended early in evaluation of the pregnant cardiac arrest patient [1,11]. In this case, ultrasound was performed in ED to determine the need for cesarean delivery. Cesarean delivery was performed when the patient declared hemodinamically stabilize after six hours admission to the ICU. The ACLS guidelines for defibrillation, intubation and pharmacologic agents are also recommended in the pregnant by the recent resuscitation guidelines of UK and American Heart Association (AHA) [2,10]. In this case, defibrillation and medications were given in the usual manner as recommended in the ACLS guidelines.

In conclusion, we emphasize that understanding the causes of cardiac arrest during pregnancy, its early recognition and prompt resuscitation by recent ACLS guidelines may decrease both maternal and fetal morbidity or mortality. Defibrillation and doses of medication used in resuscitation of the maternal collapse are the same as those required for other adults with cardiac arrest. Coordinated integration of prehospital, emergency medicine, obstetrics, anesthesiology, pediatric, intensive care medicine or rehabilitation care, also widespread training of rescue team have important impacts on improving survival of both mother and baby.
